# Syndrome d'histoplasmose oculaire présumée (POHS)

**DOI:** 10.11604/pamj.2014.18.268.4692

**Published:** 2014-08-02

**Authors:** Mina Laghmari, Omar Lezrek

**Affiliations:** 1 Faculté de Médecine et de Pharmacie de Rabat, Université Mohammed V Souissi, Rabat, Maroc

**Keywords:** Syndrome, histoplasmose oculaire, POHS, syndrome, ocular histoplasmosis, POHS

## Image en medicine

Un patient âgé de 40 ans, marocain autochtone, sans antécédents particuliers, présente une baisse de l'acuité visuelle (AV) avec métamorphopsies et scotome central de l'oeil droit, apparus depuis quelques semaines. L'examen de l'oeil droit trouve une AV à 1/10 Parinaud 14. L'examen biomicroscopique ne trouve pas de Tyndall de chambre antérieure ni de hyalite. L'examen du fond d'oeil droit met en évidence un décollement sérohémorragique maculaire avec des lésions atrophiques profondes de moins d'un quart de diamètre papillaire, en nasal de la papille et en parafovéolaire inférieur correspondant à des histospots (A). L'examen de l'oeil gauche est normal avec une AV à 10/10 Parinaud 1,5. Cet aspect est évocateur du syndrome d'histoplasmose oculaire Présumée (POHS). L'angiographie fluorescéinique montre au temps précoce un lacis vasculaire profond correspondant à des néovaisseaux sous rétiniens (B), et au temps tardif une diffusion intense des néovaisseaux sous rétiniens avec effet fenêtre au niveau des histospots (C). Décrit initialement par Woods et Whalen puis par Gass, le POHS associe une triade incluant un décollement sérohémorragique de la macula, des cicatrices choriorétiniennes atrophiques de l'aire maculaire ou de la périphérie rétinienne (histospots) et des cicatrices péripapillaires sans aucun signe inflammatoire oculaire. La néovascularisation choroïdienne se développe à partir des cicatrices et siège habituellement dans la région parafovéolaire. Aux USA, la fréquence des réactions positives à l'histoplasmine a fait attribuer cette affection à*histoplasmacapsulatum*. En Europe et en Afrique les cas d'affections à *histoplasmacapsulatum* sont rares; on parle d'histoplasmose oculaire présumée.

**Figure 1:**
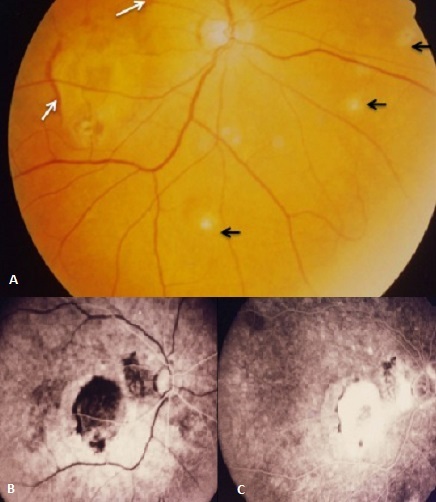
(A): rétinographie de l’œil droit; (B):Temps précoce de la séquence angiographique; (C): Temps tardif de la séquence angiographique

